# Dr. Marsden

**Published:** 1836-04

**Authors:** 


### DR. MARSDEN.

We observe the announcement of the recent death of Dr. Marsden, of Nottingham, at an advanced age.

